# Double stent insertion for combined malignant airway and superior vena cava obstruction

**DOI:** 10.1097/MD.0000000000015777

**Published:** 2019-05-24

**Authors:** Jing-Yan Ren, Chi Cao, Yu-Fei Fu, Hong-Tao Du

**Affiliations:** a Department of Intensive Care Unit, Zaozhuang Hospital of Beijing Traditional Chinese Medical University, Zaozhuang; b Department of Radiology, Xuzhou Central Hospital, Xuzhou, China.

**Keywords:** airway, double, stent, superior vena cava

## Abstract

The present study sought to assess the feasibility and effectiveness of double stent insertion as a means of managing combined malignant airway and superior vena cava (SVC) obstruction (CMASO).

From July 2010 to January 2018, twelve consecutive patients with CMASO were treated by double stent insertion (airway and SVC stents) in our centers. We assessed data pertaining to both technical and clinical success rates, as well as to long-term patient outcomes.

The use of double stents (12 airway stents and 19 SVC stents) was technically successful in all study subjects, with a 0 to 92 days period between the 2 stent insertions (mean 27.8 days). Patients did not show evidence of any procedure-related complications. Mean patient Hugh-Jones grades improved from 4.4 ± 0.5 before inserting the airway stent down to 1.2 ± 0.4 following this insertion (*P* < .001). Mean SVC pressure was reduced from 17.5 ± 2.8 mm Hg before the stent insertion down to 6.7 ± 1.4 mm Hg following this insertion (*P* < .001). Fifty-six days after insertion, a single patient experienced re-obstruction of their SVC stent. All patients died within the follow-up period, with a median survival time of 113 days for these 12 patients.

This double stent insertion protocol is both effective and safe as a means of offering palliative care to those with CMASO.

## 1 Introduction

Malignant airway or superior vena cava (SVC) obstructions may occur as a consequence of cancers of the lung, esophagus, or mediastinum.^[1–4]^ Stent insertion is a commonly employed palliative strategy in patients with inoperable malignant airway or SVC obstruction.^[1–4]^ After stent insertion, patients can undergo subsequent chemotherapy or radiotherapy to prolong their survival time.^[5]^ However, because of close proximity of the SVC and the airway, both may be simultaneously obstructed by a tumor.^[6–8]^ Treating patients that have combined malignant airway and SVC obstruction (CMASO) is challenging. At present, there are few reports regarding the management of CMASO.^[6–8]^

This study, therefore, sought to determine how feasible and effective double stents insertion was for the management of CMASO.

## 2 Patients and methods

This single-center retrospective study was approved by our Institutional Review Board. The requirement of written informed consent was waived.

## 3 Patients

Between July 2010 and January 2018, 12 consecutive patients (7 male, 5 female; mean age, 64.3 ± 8.2 years; range, 49–76 years) with CMASO were treated by double stent insertion (airway and SVC stents) in our centers. Patients’ baseline data were demonstrated in Table 1.

**Table 1:**
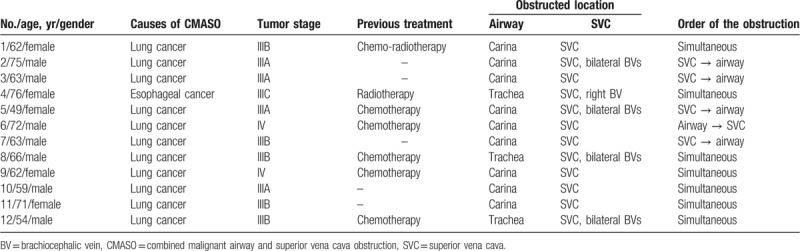
Patients’ characteristics.

Inclusion criteria for these patients were:

(1) a confirmed diagnosis of CMASO, and
(2) inoperable cases.

We excluded any patients that had major dysfunctions of heart, kidney, or lung tissue, or who had high levels of coagulation.

## 4 Diagnosis

CMASO causes were lung cancer (n = 11) and esophageal cancer (n = 1). The primary tumors were identified based on appropriately conducted biopsies and/or endoscopic and bronchoscopic findings. Airway obstruction was diagnosed on the basis of symptoms, patient history, and a thoracic computed tomography (CT) scan. Obstruction location was evaluated by thoracic CT before treatment. Diagnosis of SVC obstruction was based on the same information as the airway obstruction, with the addition of intraoperative venography findings. The extent of SVC obstruction was evaluated by thoracic CT and intraoperative venography findings.

### 4.1 Airway and SVC stents

Airway stents used in the present study were bare self-expanding metallic stents (Micro-Tech, Nanjing, China). Two primary stent formats were utilized depending on the obstruction location (see Table 1). Tubular stents were utilized in those patients with tracheal obstructions, and Y-shaped stents were utilized in those with carinal obstructions.

The SVC stents used in the present study were also bare self-expanding metallic stents, including the Sinus-XL (OptiMed, Ettlingen, Germany), Zilver (Cook, IN), and Smart (Cordis, FL) stents.

For all stents used, both the proximal and distal stent margins were a minimum of 10 mm longer than those same margins on the obstruction, and the stent diameter was a minimum of 2 mm greater than that of the corresponding airway or SVC.

### 4.2 Airway stent insertion

**Figure 1:**
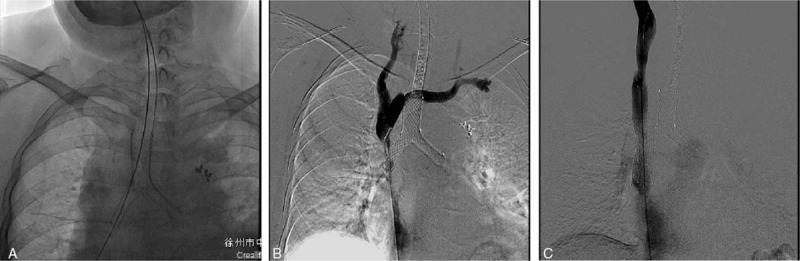
A 62-yr-old woman with CMASO underwent airway and SVC stents insertion. (A) A Y-shaped airway stent was inserted first. (B) SVC venography demonstrated the obstruction site of SVC. (C) SVC was patent after SVC stent insertion. CMASO = combined malignant airway and SVC obstruction, SVC = superior vena cava.

Three interventional radiologists performed the airway stent insertion procedure in all patients under fluoroscopic guidance (Fig. 1).

Airway stent insertion was performed under the general anesthesia. For those patients requiring a tubular stent, a 0.035-inch guidewire (Terumo, Tokyo, Japan) and a 4 Fr VER catheter were inserted through the mouth and into a main bronchi, at which point the initial guidewire was switch with a stiff guidewire of the same measurements (Cook). The 14 Fr stent delivery system was then inserted along the stiff guidewire, with the stent positioned so as to cover the site of the obstruction. For patients requiring Y-shaped stents, 2 stiff 0.035-inch guidewires were bilaterally inserted into the bronchi, after which a 27 Fr stent delivery system was inserted along these wires, allowing the stent to be deployed at the site of the obstruction. The oxygen catheter was removed following the completion of the stent insertion.

Following completion of the insertion process, patients underwent nebulizer treatment once daily for a total of 3 days in an effort to loosen sputum and to thereby minimize ant-associated chest pain. Within 1 week of the stent insertion procedure, all patients received a thoracic CT and underwent a complete clinical examination to ensure that the stent was properly localized.

### 4.3 SVC stent insertion

SVC stents were inserted into patients via a right femoral vein approach using fluoroscopic guidance (Fig. 1). Obstruction of SVC and bilateral brachiocephalic veins was treated via unilateral stent insertion and recanalization of right brachiocephalic vein. At the site of the obstruction, both the 4 Fr VER catheter and 0.035-inch guidewire were inserted, with venography being used to confirm the location of the obstruction. Once confirmed, a stiff 0.035-inch guidewire was inserted across the obstruction site, after which a 7-10 Fr stent delivery system was inserted along this guidewire, with the stent being deployed so as to cover the site of the obstruction. In order to ensure correct SVC patency, venography was conducted at the end of the insertion process, measuring the pressure gradient between the proximal and distal ends of the obstructed site and the stent so as to compare these values to those prior to stent insertion.

For 3 days following stent insertion, patients received 5000 IU subcutaneous low-molecular-weight heparin every 12 hours, after which they received oral warfarin sodium. The international normalized ratio was maintained between 2 and 3.

### 4.4 Order of the stents insertion

For patients with simultaneous obstructions of the airway and SVC, airway stents were inserted first before SVC stent insertion. In patients with non-simultaneous obstructions, stents were inserted in the order in which obstructions arose.

### 4.5 Study endpoints

Technical airway stent success was defined on the basis of correctly placing the stent so that it was positioned across the target obstruction site.^[9–11]^ Clinical success of airway stent insertion was defined based on the patient experiencing a minimum of 1-grade improvement in their Hugh-Jones classification score.^[9–11]^ For SVC stents, technical insertion success was defined on the basis of correcting placing the stent so as to cross the site of the obstruction while maintaining a pressure gradient <10 mm Hg between proximal and distal stent ends.^[12,13]^ Clinical success for the insertion of SVC stents was defined on the basis of substantial reduction in or elimination of the SVC syndrome in treated patients.^[12,13]^ Duration of survival was monitored for all patients.

All patients were monitored over time using a combination of CT scans and telephone-based interviews. Patients underwent a thoracic CT scan after 1 month, and then again each 2 to 3 months following stent insertion. Patients also received a telephone call-based follow-up interview every 2 months in order to assess the overall general condition of the patient. These follow-up activities were continued until the time of patient death. Survival duration was the primary study endpoint, while rates of complications and re-obstruction served as secondary endpoints

### 4.6 Statistical analysis

Continuous variables are given as mean ± standard deviation. To compare these values before and after stent insertion, paired *t* tests were used. Kaplan–Meier curves were used to assess survival time. A 2-tailed *P* < .05 was considered to be significant. SPSS v16.0 (SPSS, Inc, Chicago, IL) was used for all statistical analyses.

## 5 Results

### 5.1 Technical success

The insertion of airway stents was technically successful in all 12 patients. Eleven patients experienced technical success of airway stent insertion at the first attempt. One patient (Patient 5) failed to undergo Y-shaped stent insertion at the first attempt because the 27 Fr stent delivery system could not pass through her larynx. Therefore, we inserted a 16 Fr stent delivery system loaded L-shaped stent at trachea and right main bronchus for this patient. As for the tubular stent, the diameter ranged from 16 to 18 mm and the length ranged from 50 to 60 mm. The body diameter of the Y-shaped stents ranged from 18 to 20 mm, and length of the body ranged from 30 to 40 mm. Bronchial limb diameters ranged from 11 to 12 mm, while the length of these limbs ranged from 15 to 20 mm. For the L-shaped stent, the body diameter and length were 18 and 30 mm, respectively, while those of the bronchial limbs were 12 and 20 mm, respectively.

All patients underwent technically successful SVC stent insertion. In total, 19 SVC stents were placed in these 12 patients. SVC stent diameter ranged from 12 to 18 mm, while the length ranged from 60 to 80 mm. Mean pressure gradients were lowered in patients from 17.5 ± 2.8 mm Hg before inserting the stent down to 6.7 ± 1.4 mm Hg following this insertion (*P* < .001).

Table 2 summarizes patient stent insertion order. The time between stent insertions ranged from 0 to 92 days (mean 27.8 days). Patients did not suffer any significant complications relating to the insertion procedures.

**Table 2:**
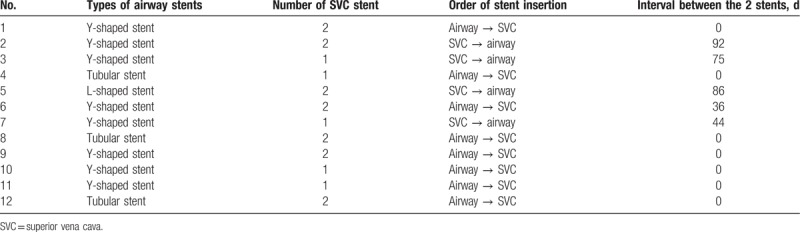
Treatment details.

### 5.2 Clinical success

All patients exhibited the criteria for clinical success following stent insertions (Table 3). Patients experience a mean Hugh-Jones grade reduction from 4.4 ± 0.5 to 1.2 ± 0.4 following the insertion of the airway stent (*P* < .001). SVC syndrome was completely relieved after SVC stent insertion in all patients.

**Table 3:**
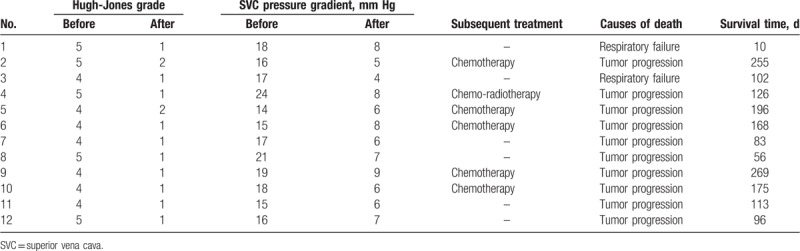
Effectiveness and outcomes.

### 5.3 Follow-up

**Figure 2:**
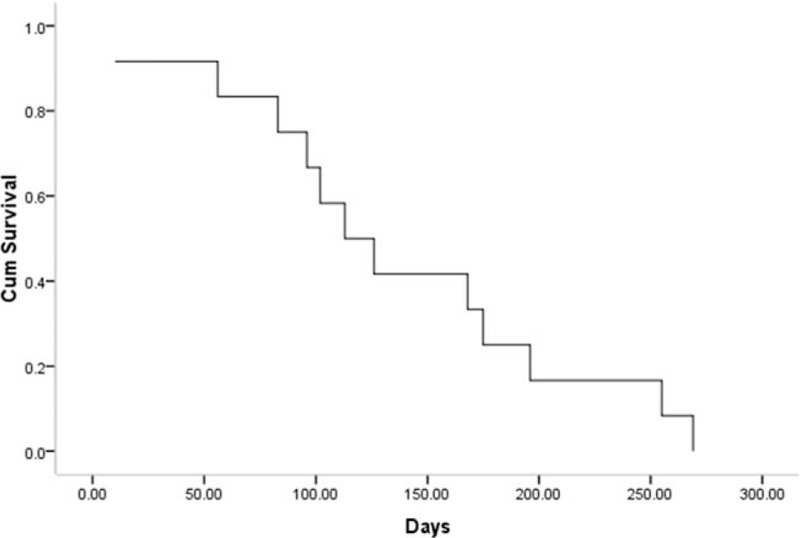
Kaplan–Meier curves of survival after double stents insertion.

All patients were followed for 10 days – 9 months (mean 4.9 ± 2.6 months). Six patients underwent subsequent anticancer treatment after stent insertion. One patient showed signs of re-obstruction of the SVC stent 56 days after stent insertion and this patient was managed with chemotherapy. All patients died within the follow-up period from either tumor progression (n = 10) or respiratory failure (n = 2). For the 12 patients included in the present study, the median survival time following this double stent insertion procedure was 113 days (95% confidence interval, 72.3–153.7 days, Fig. 2).

## 6 Discussion

The present study was conducted so as to assess the efficacy and feasibility of double stent insertion in those suffering from CMASO. We achieved both technical and clinical success through this approach in all patients, and the survival times for patients enrolled in this study was promising.

Individuals suffering from malignant airway or SVC obstructions typically serve as poor candidates for surgical tumor resection as such obstructions typically arise when tumors are of a very advanced stage.^[1–4]^ In addition, airway obstruction can cause severe dyspnea, which also may cause patients’ death.^[1,2]^ SVC obstruction usually causes dyspnea, facial or neck swelling, and arm swelling. These symptoms usually reduce patients’ quality of life for the remaining period of time during which they survive.^[3,4]^ Patients with CMASO may have more serious illness condition than patients with only airway or SVC obstruction.

Treatment of CMASO is challenging. While patient care for this condition is typically based upon a combination of radio- and chemotherapy, the efficacy of these approaches is limited and the use of this approach thus remains debated.^[11–13]^ As such, the first line approach for care is now stent insertion, as such stent insertion provides urgent relief of symptoms without interfering with subsequent anticancer therapies.^[9–13]^

In the present case series, all of the stents used to treat airway obstructions were of a self-expanding metallic variety, as has been used in treating airway obstruction, airway-alimentary tract fistula, or airway stump fistula.^[9–11,14,15]^ The most used airway stents for airway obstruction are tubular and Y-shaped stents.^[9–11]^ It is also possible to insert self-expanding metallic airway stents with fluoroscopic guidance, offering those positioning the stent a complete panoramic view of the airway in order to optimally position and release the stent at the site of the obstruction.^[11]^

Between 10% and 60% of patients with SVC obstructions exhibit SVC and bilateral brachiocephalic vein obstruction.^[3,12,13]^ At the present case series, 2 patients (40%) presented with obstruction of SVC and bilateral brachiocephalic veins, and this result is comparable to the previous studies.^[3,12,13]^ However, treatment of obstruction of SVC and bilateral brachiocephalic veins is not complicated, and unilateral stent insertion can achieve clinically successful outcomes.^[12,13]^

Table 4 demonstrates outcomes from previous studies assessing double stent insertion for treating CMASO. Fukuda et al^[6]^ and Morales et al^[7]^ respectively described a case of double stents insertion for simultaneous airway and SVC obstruction. However, Kapadia et al^[8]^ described a case of secondary SVC obstruction after airway stent insertion, and the SVC stent insertion acted as a salvage.

**Table 4:**
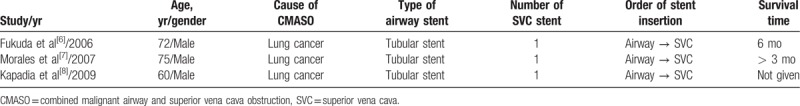
Reported cases of double stents insertion for CMASO.

In the present case series, 7 patients had simultaneous airway and SVC obstruction. The order of double stents insertion was airway stent followed by SVC stent. When inserted, airway stents can both overcome airway obstructions and prevent further obstructions caused by the SVC stent. Median survival of these 12 patients following stent insertions was 113 days, which is similar to other studies of double stents insertion for CMASO.^[6,7]^

There are certain limitations to the present study, including the fact that it is retrospective in nature opening it to the risk of selection bias. This study also has a small sample size, and so results must be interpreted with caution. However, because CMASO is a rare condition, we feel that these results offer sufficient insights so as to highlight the feasibility and effectiveness of this technique. This study also lacks a control group, and as such these results cannot be compared to those of other treatment strategies, such as the insertion of a single stent. Final, the computational fluid dynamics (CFD) analysis was not used in this study. At present, CFD analysis was widely used in evaluating the hemodynamic or flow dynamic parameters for patients with vessel or airway stenosis.^[16–18]^ Further studies should contain the CFD parameters to evaluate the effectiveness of stent insertion.

In summary, our research provides evidence that double stents can be safely and effectively inserted into patients suffering from CMASO, thus offering them palliative care, although further clinical trials will be needed to validate these findings. We further recommend utilizing self-expanding metallic stents for both airway and SVC insertion.

## Author contributions

**Conceptualization:** Yu-Fei Fu, Hong-Tao Du.

**Data curation:** Jing-Yan Ren, Hong-Tao Du.

**Formal analysis:** Jing-Yan Ren.

**Investigation:** Chi Cao.

**Methodology:** Chi Cao, Hong-Tao Du.

**Project administration:** Yu-Fei Fu.

**Resources:** Chi Cao.

**Software:** Yu-Fei Fu, Hong-Tao Du.

**Supervision:** Hong-Tao Du.

**Validation:** Yu-Fei Fu.

**Writing – original draft:** Jing-Yan Ren.

**Writing – review and editing:** Yu-Fei Fu.
